# On the mechanism of autoinhibition of the RhoA-specific nucleotide exchange factor PDZRhoGEF

**DOI:** 10.1186/1472-6807-9-36

**Published:** 2009-05-21

**Authors:** Meiying Zheng, Tomasz Cierpicki, Ko Momotani, Mykhaylo V Artamonov, Urszula Derewenda, John H Bushweller, Avril V Somlyo, Zygmunt S Derewenda

**Affiliations:** 1 Department of Molecular Physiology and Biological Physics, University of Virginia, PO Box 800736, Charlottesville, Virginia. 22908-0736, USA; 2 Present address : Monsanto Company, 800 North Lindbergh Boulevard, St Louis, MO 63167

## Abstract

**Background:**

The Dbl-family of guanine nucleotide exchange factors (GEFs) activate the cytosolic GTPases of the Rho family by enhancing the rate of exchange of GTP for GDP on the cognate GTPase. This catalytic activity resides in the DH (Dbl-homology) domain, but typically GEFs are multidomain proteins containing other modules. It is believed that GEFs are autoinhibited in the cytosol due to supramodular architecture, and become activated in diverse signaling pathways through conformational change and exposure of the DH domain, as the protein is translocated to the membrane. A small family of RhoA-specific GEFs, containing the RGSL (regulators of G-protein signaling-like) domain, act as effectors of select GPCRs *via *Gα_12/13_, although the molecular mechanism by which this pathway operates is not known. These GEFs include p115, LARG and PDZRhoGEF (PRG).

**Results:**

Here we show that the autoinhibition of PRG is caused largely by an interaction of a short negatively charged sequence motif, immediately upstream of the DH-domain and including residues Asp706, Glu708, Glu710 and Asp712, with a patch on the catalytic surface of the DH-domain including Arg867 and Arg868. In the absence of both PDZ and RGSL domains, the DH-PH tandem with additional 21 residues upstream, is 50% autoinhibited. However, within the full-length protein, the PDZ and/or RGSL domains significantly restore autoinhibition.

**Conclusion:**

Our results suggest a mechanism for autoinhibition of RGSL family of GEFs, in which the RGSL domain and a unique sequence motif upstream of the DH domain, act cooperatively to reduce the ability of the DH domain to bind the nucleotide free RhoA. The activation mechanism is likely to involve two independent steps, i.e. displacement of the RGSL domain and conformational change involving the autoinhibitory sequence motif containing several negatively charged residues.

## Background

Rho (***R***as-***h***omology) cytosolic GTPases function as molecular switches that, in the GTP-bound form, interact with a multitude of effectors that exert control over cytoskeletal elements, gene transcription, and other biological phenomena [1-3]. Spatial and temporal control over these GTPases is exercised by GEFs (guanine nucleotide exchange factors), which load up GTP and activate cognate GTPases, and by GAPs (GTPase activating proteins) which are required by the GTPase for efficient hydrolysis of GTP to GDP [4,5]. Most of Rho GEFs belong to the Dbl-homology family of large, multidomain proteins [6]. There are approximately 70 of these proteins in the human proteome, some highly specific and some activating indiscriminately two or more different Rho GTPases [5,7]. The catalytic step is executed by the Dbl-homology (DH) domain, often assisted by a pleckstrin-homology (PH) domain, which is invariably located immediately downstream of the DH domain [6,7]. The DH domain, either alone or synergistically with the PH-domain, binds the cognate GTPase in its nucleotide-free form. Upon release, the GTPase forms a biologically active complex with the more abundant GTP nucleotide [8]. The GEFs are thought to be inactive in their nascent form due to a 'folded', or 'closed' conformation, in which specific domains or motifs outside the DH-PH tandem bind to those surfaces on the DH domain which are involved in the binding of the GTPase [7]. The activation of specific GEFs requires extra- or intracellular stimuli that directly or indirectly induce conformational changes in GEFs leading to release of autoinhibition and expression of full catalytic potential. While this paradigm may be generally conserved, details vary depending on the architecture of a particular GEF.

Recently, structural studies elucidated the mechanism of autoinhibition in several Dbl-homology GEFs. The proto-oncogene product Vav, which activates Rac1, is autoinhibited by an N-terminal, helical extension of the DH-domain which lies in the GTPase interaction site. This autoinhibition is relieved by Src-mediated phosphorylation of Tyr174, which is in the center of the autoinhibitory helix [9]. In Asef, a Rac-specific exchange factor, the activity of its DH domain is suppressed through an intramolecular interaction with an SH3 domain, found immediately upstream of the DH module [10,11]. It is possible that a similar mechanism is also in operation in other GEFs that show a similar architecture of SH3-DH-PH modules. In contrast, the Rho-specific p63RhoGEF contains no identifiable domains other than the DH-PH tandem, and the autoinhibition is mediated by a conserved, α-helical C-terminal extension of the PH domain [12]. Analogous extensions are found in Trio and Kalirin, and the autoinhibition mode of these GEFs may be very similar to that of p63RhoGEF [12].

A family of three RhoA-specific GEFs act downstream of the Gα_12/13_-coupled receptors (GPCRs). They are: p115RhoGEF [13], PDZRhoGEF, henceforth referred to as PRG [14], and LARG [15,16]. This family is distinguished by the presence of the RGSL (regulator of G-protein signaling-like) domain, upstream of the DH-PH tandem. Further, both PRG and LARG contain additional N-terminal extensions which include a PDZ (*P*SD-95, Disc-large, ZO1) domain. Finally, downstream of the DH-PH tandem, all three GEFs contain a ~400 residue long, largely unstructured region which include a coiled-coil fragment that mediates homo- and heterodimerization [17].

There is considerable evidence that Gα_12/13 _activates the RGSL-family of Dbl-homology GEFs *via *direct interaction with the RGSL domain. Crystal structures of these domains from both p115 and PRG have been determined [18,19], and additionally the structure of the complex of the p115 RGSL domain with the Gα_12/i3 _chimera elucidated the nature of the interaction of both proteins [20]. However, this did not explain how RGSL-domain containing GEFs are activated. Further, it appears that mere physical interaction does not necessarily result in up-regulation of GEF activity. For example, the RGSL domain of p115 has been shown to bind to both Gα_12 _and Gα_13_, but only Gα_13_, stimulates the GEF activity for p115RhoGEF and non-phosphorylated LARG [21]. Gα_12 _will stimulate LARG when the latter is phosphorylated, although it is not known why [22]. An excellent review of the interactions of RGSL-family GEFs with G proteins was published recently [23].

There is also evidence that the PDZ domain may be important for the activation of LARG and PRG. Typically, these relatively small domains are expected to play a role in targeting proteins to large membrane-associated protein complexes [24-26]. However, there is evidence that interactions mediated by the PDZ domains of both LARG and PRG may also activate their nucleotide exchange function. For example, plexin-B family of receptors bind the PDZ domains of both LARG and PRG leading to RhoA activation [27-29]. It has also been reported that the lysophosphatidic acid receptors, LPA1 and LPA2, bind the PDZ domain of PRG, and that this interaction leads to activation of the RhoA pathway in HEK293 cells [30]. The same PDZ domain was also shown to interact with the microtubule associated protein 1 (MAP1) light chain and this interaction modulated the GEF activity [31]. Finally, the PDZ domain of LARG was shown to bind to CD44 (major hyaluronan receptor), also upregulating RhoA [32].

Since the mechanism of autoinhibition of the RGSL-family of GEFs has not been elucidated, it is difficult to speculate how the interactions mediated by RGSL and PDZ domains may lead to activation of LARG or PRG, and if they act synergistically or through alternative and independent mechanisms. In the present study we investigated the question of the molecular basis of autoinhibition of the RGSL family of GEFs. We find that the recombinant fragment encompassing residues 37–1081 and including all four key domains (PDZ, RGSL, DH and PH) of PRG is indeed autoinhibited, and shows only ~15% of the intrinsic catalytic activity of the isolated DH-PH tandem. Unexpectedly, the removal of the PDZ domain, or of a fragment containing both the PDZ and RGSL domains, has limited effect on the catalytic rates, which are 18% and 35% of that of the isolated DH-PH tandem, respectively. We identify an autoinhibitory element within the sequence immediately upstream of the DH-PH tandem, and we trace the autoinhibition of PRG primarily to an interaction between a negatively charged sequence motif, including Asp706, Glu708, Glu710 and Asp712, and a positive patch on the DH domain that is involved in the recognition and binding of RhoA. Our results reveal a complex autoinhibitory mechanism and suggest the possibility that in addition to relief of autoinhibition through protein-protein interactions, actual up-regulation of the intrinsic nucleotide exchange activity of GEFs by interacting partners may constitute a hitherto unrecognized regulatory mechanism.

## Results and discussion

### Identification of an autoinhibitory element in the RGSL-DH linker region

**Figure 1:**
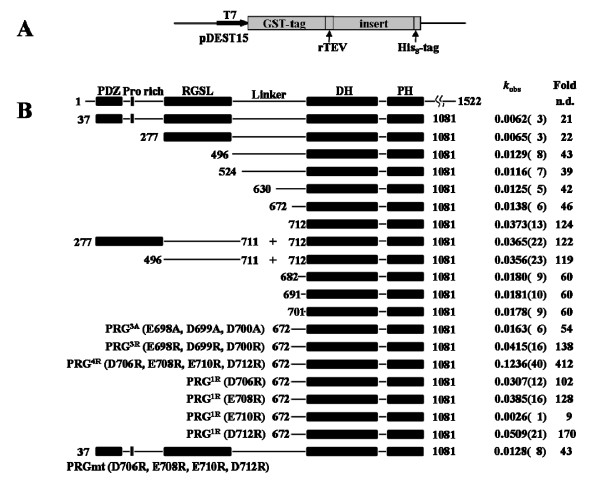
**Multidomain fragments and mutants of PRG generated in this study and their functional characterization; (A) diagrammatic representation of the expression vector used for all constructs; (B) The constructs used in this study, the *k*_*cat *_values and fold-enhancement of nucleotide exchange compared to uncatalyzed RhoA**.

Multidomain fragments of the human PRG were expressed in *E. coli *as described in Materials and Methods. We were not able to express the full-length protein encompassing residues 1–1522, but we were able to express the core fragment containing the four functional domains, i.e. PDZ, RGSL, DH and PH, with the connecting linkers, i.e. residues 37–1081. Initial experiments showed that constructs containing a single His_6 _tag at the N-terminus resulted in expression of heterogeneous mixtures of products, probably due to incomplete translation. The problem was effectively solved by replacing the N-terminal His_6 _tag with a GST tag and adding a His_8 _tag at the C-terminus, so that full-length products could be readily purified using tandem-affinity chromatography. Figure 1 shows a diagram of the expression plasmid, the constructs which were expressed and purified in this study and the results of the nucleotide exchange assay for each, while figure S1 (Additional File 1) illustrates the purity of the final protein samples. The shortest fragment comprised only the DH-PH tandem (PRG^712–1081^), previously shown by us to have maximum catalytic efficiency on recombinant RhoA (residues 1–181) with a nucleotide exchange enhancement of app. 130-fold compared to spontaneous rates [33].

**Figure 2:**
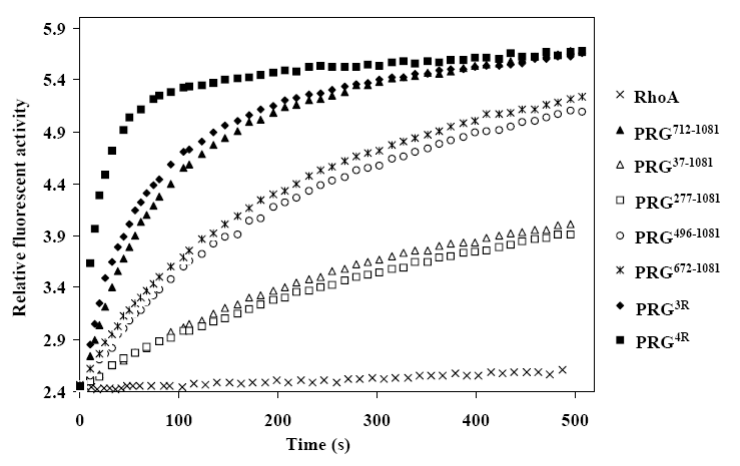
**Representative results of the nucleotide exchange assay**. Only half of experimental points are visualized in the graph. For other details refer to Materials and Methods.

As judged by the fluorescence assay (see figure 2 for representative raw data), all constructs longer than the DH-PH tandem exchanged GDP for mant-GTP far less efficiently, consistent with the notion that structural elements located upstream of the DH-PH tandem inhibit the catalytic function. The construct containing all four domains (PDZ-RGSL-DH-PH, or PRG^37–1081^) enhanced the nucleotide exchange only 21-fold, and the RGSL-DH-PH construct (PRG^277–1081^), lacking the PDZ domain, showed essentially identical activity on RhoA, consistent ~80% autoinhibition. This baseline activity is comparable to the levels observed for other GEFs: for example, the C-terminal extension on p63RhoGEF confers 90% inhibition [12]; for Dbl, the full length protein retains about 10% of the activity of the isolated DH-PH domain [34]; full length TIM enhances nucleotide exchange on RhoA 5-fold, and the removal of the N-terminal 22 residues increases the effect to 30-fold [35]. A recent study elegantly demonstrated that the baseline activity of Vav1 is due to an equilibrium between the inhibited, ground state, and an excited state which is transiently active, highlighting the importance of protein dynamics in cell regulation [36].

Unexpectedly, the removal of the RGSL domain (PRG^496–1081^) did not relieve autoinhibition, and the resulting protein showed only 33-fold enhancement in nucleotide exchange on RhoA, i.e. ~65% autoinhibition. Thus, we concluded that key elements responsible for autoinhibition reside within the 200 amino acid linker sequence between the RGSL and DH domains. Secondary structure prediction suggests that the linker sequence has propensity to form localized helical structures, although more than 50% of the sequence is predicted to harbor unstructured regions (not shown). We were able to express the linker region (residues 476–711) and a CD (circular dichroism) spectrum (not shown) was consistent with a random coil structure. Given that the sequence gave no clue as to the identity of the functionally important fragment, we expressed additional, progressively shorter constructs, i.e. PRG^524–1081^, PRG^630–1081^, PRG^672–1081 ^and PRG^691–1081^. All retained a significant level of autoinhibition. We then measured the nucleotide exchange rate on RhoA in the presence of both the isolated DH-PH tandem (i.e. PRG^712–1081^), and the isolated fragment containing the RGSL domain with the C-terminal linker (i.e. PRG^277–711^), or just the recombinant linker (i.e. PRG^496–711^). Both experiments gave results identical to those obtained for the DH-PH tandem alone, indicating that the neither the RGSL domain, nor the RGSL domain with its C-terminal extension are capable of interacting with the DH-PH tandem in the absence of a covalent link.

### Chemical shift assignment of DH-PH

**Figure 3:**
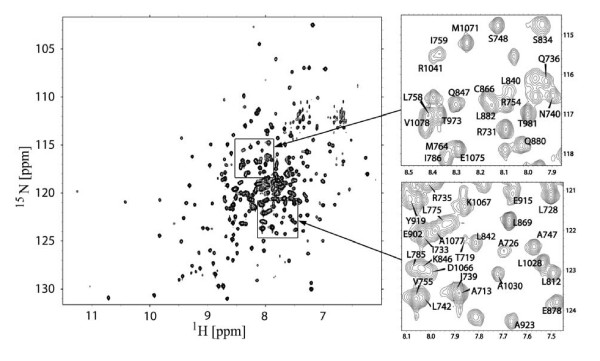
**The 800 MHz ^1^H-^15^N TROSY-HSQC spectrum of the 0.3 mM DH-PH tandem at 30°C; assignment is shown for two selected fragments of the spectrum**.

To probe the molecular mechanism of PRG autoinhibition we used NMR spectroscopy. The ^1^H-^15^N TROSY-HSQC spectrum of triple-labeled, DH-PH tandem (PRG^712–1081^) recorded at 800 MHz is shown in figure 3. Due to the large size of the protein (43 kDa) the spectrum shows considerable signal overlap. To obtain chemical shift assignment for the DH-PH resonances, we first recorded separate spectra for isolated DH and PH domains. We designed the corresponding constructs based on the crystal structure of the DH-PH/RhoA complex [37], so that the domains were separated between residues 932 and 933, within the interdomain α-helix. The two isolated domains, i.e. DH (residues 712–932) and PH (residues 933–1081), yield spectra of sufficient quality to allow for backbone assignment using TROSY-based triple resonance experiments. We achieved nearly complete, 92% assignment of chemical shifts for the isolated DH domain, and a 70% assignment for the PH domain, which required 0.6 M NaCl to reduce protein aggregation and consequent peak broadening.

When we compared the spectra of the DH-PH tandem with the spectra of the isolated domains, we found that a vast majority of signals were only slightly shifted, allowing us to use the assignment for the isolated domains to directly assign the chemical shifts for the tandem. In addition, we used the sequential connectivities derived from HNCA and ^1^H-^15^N HSQC-NOESY experiments measured for the tandem. Overall, we assigned 72% of backbone amides in the DH-PH tandem: 84% for the DH domain but only 55% for the PH domain, due to resonance broadening.

### Interactions of the autoinhibitory element with the DH domain and with RhoA

**Figure 4:**
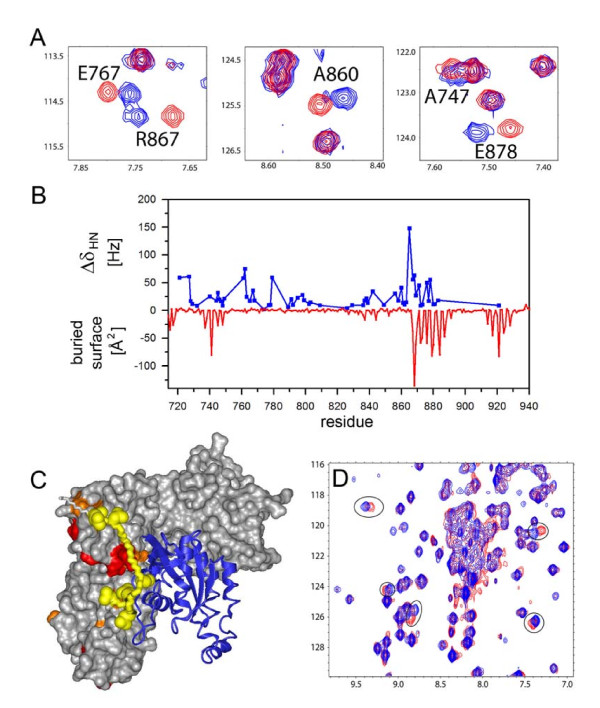
**The fragment containing residues 672–712, upstream of the DH-PH tandem, interacts with residues within the DH domain and with RhoA in the binary complex**. **(A) **comparison of 900 MHz ^1^H-^15^N TROSY-HSQC spectra of DH-PH (blue) and PRG^672–1081 ^(red) showing chemical shift changes within DH domain induced by the linker; only select fragments of the spectrum are shown for clarity **(B) **a graph showing the magnitude of linker induced chemical shift changes (Δσ_HN _[Hz]) within the DH domain (blue) compared to the surface area of DH residues buried upon formation of the complex with RhoA (red); Δσ_HN _[Hz] were calculated as a differences between chemical shifts for PRG^672–1081^and DH-PH; the surface buried upon complex formation was calculated using the crystal structure of DHPH-RhoA complex and the program MOLMOL [38]; **(C) **crystal structure of the DH-PH/RhoA complex (PDB code 1XCG), showing the residues within the DH domain with the largest amide chemical shift changes induced by the linker (>50 Hz, red; 25 – 50 Hz, orange); the hypothetical position of the linker, postulated on the basis of chemical shift perturbations, is shown in yellow; side chains of acidic residues within the linker are shown in space filling representation; RhoA is shown as blue ribbon; **(D) **comparison of 600 MHz ^1^H-^15^N TROSY-HSQC spectra of RhoA in complex with DH-PH (blue) and PRG^672–1081 ^(red); several RhoA amides with chemical shifts perturbed by the presence of the linker are circled.

To assess if the DH-PH tandem is structurally perturbed by the presence of the N-terminal extensions (i.e. the RGSL-DH linker fragments), in the autoinhibited constructs PRG^630–1081 ^and PRG^672–1081^, we studied both proteins in solution using heteronuclear NMR. Despite the high molecular weight of the proteins (>43 kDa), protein deuteration allowed us to record high quality ^1^H-^15^N TROSY-HSQC spectra. We compared them to those obtained by us for the PRG^712–1081 ^fragment (i.e. the isolated DH-PH tandem). All three spectra are similar, except for additional peaks due to the unstructured residues from the linker in the longer constructs (data not shown). The peaks associated with the DH-PH tandem amides were superimposable for the two longer constructs, indicating that the linker does not significantly perturb the structure of the tandem; this observation is consistent with the nucleotide exchange assay. However, a detailed analysis of the ^1^H-^15^N TROSY-HSQC spectra measured at 900 MHz for PRG^672–1081 ^clearly identified a group of amides with markedly perturbed chemical shifts compared to PRG^712–1081 ^(Fig. 4A) [38]. We assigned these resonances based on the assignment obtained for PRG^712–1081 ^and found that the corresponding residues are within the DH domain and cluster into three segments: 712–745, 761–779 and 842–878 (see Fig. 4B). The first cluster is located at the very N-terminus of the DH domain and the changes observed in this region are likely to be caused simply by the presence of additional upstream residues. However, the most interesting changes are observed for the third cluster of residues, which overlaps with a patch involved in the interaction with RhoA (Fig. 4C) [37]. This suggests that the interaction of the linker with the functionally important surfaces of the DH domain leads to steric interference and may constitute the basis of the autoinhibitory mechanism. We did not identify any peaks in ^1^H-^15^N TROSY-HSQC that would indicate the presence of structured elements within the linker sequence, arising from the linker sequestered between other structural elements; however, the crowded spectrum leaves such possibility open. We conclude that the linker-DH interaction is not accompanied by the formation of any distinct structural elements in the linker (e.g. α-helix). However, given the overall negative charge of the linker and the positive charges in the proximity of the relevant DH surfaces, the interaction of the linker with the DH domain is most likely electrostatic in nature.

In order to determine the position of the linker relative to the DH domain, we mapped residues with affected chemical shifts onto the crystal structure of the complex. Interestingly, we found that the linker could adopt a conformation, in which it can interact with RhoA. To test this we measured spectra of ^2^H,^15^N-labeled RhoA in complex with unlabeled DH-PH and PRG^712–1081^. In both cases we observed formation of tight complexes, as evidenced by slow exchange on NMR timescale. Further, we also found that the presence of the linker affects chemical shifts of several amides within RhoA (Fig. 4D). However, the magnitude of the chemical shift changes is smaller than that for the DH domain. Therefore, the interaction of the linker with RhoA is less intimate than that with the DH domain. Due to the lack of the chemical shift assignments of RhoA in the complex, the detailed analysis of perturbed residues was not possible.

### The nature of the interdomain interactions

The observation that Arg867 and Arg868 in the DH domain may be affected by interaction with the linker, prompted us to look at two negatively charged clusters in the linker: one made up of Glu698, Asp699 and Asp700, and the second which includes Asp706, Glu708, Glu710 and Asp712. These clusters are reminiscent of the so-called acidic region of Vav1, harboring the autoinhibitory helix [39]. A triple mutation E698A, D699A, D700A had little effect on the catalytic rate, as did truncation resulting in the PRG^701–1081 ^construct (see above). In contrast, a charge reversal mutant E698R, D699R, D700R (hereafter referred to as PRG^3R^) showed catalytic activity slightly above that of the isolated DH-PH tandem, suggesting complete relief of autoinhibition. Further, a charge reversal quadruple mutant of the second cluster D706R, E708R, E710R, D712R (denoted PRG^4R^) showed a significant *increase *in catalytic activity, 3.3 fold over the isolated DH-PH tandem. A possible explanation is that the kinetics of RhoA binding by the DH-PH tandem are altered, i.e. the *off *and *on *rates could be elevated, with no concomitant change in binding affinity for the nucleotide free RhoA, leading to higher turnover of nucleotide free RhoA to RhoA•GTP.

To better understand the role of individual amino acids, we generated four single-site mutants of the PRG^712–1081 ^construct, i.e. D706R, E708R, E710R and D712R (see above). The E710R mutant shows significant autoinhibition (it is actually less active in the *in vitro *assay than PRG^701–1081^), but each of the remaining mutants exhibits catalytic activity comparable to the isolated DH-PH tandem. It is therefore possible that the observed enhanced catalytic potential of the PRG^4R ^is due to additive effects of individual mutations. Interestingly, within the context of the intact four-domain construct (i.e. PRG^37–1081^) most of the autoinhibition is effectively restored, indicating that both the PDZ and RGSL domains play an important role.

**Figure 5:**
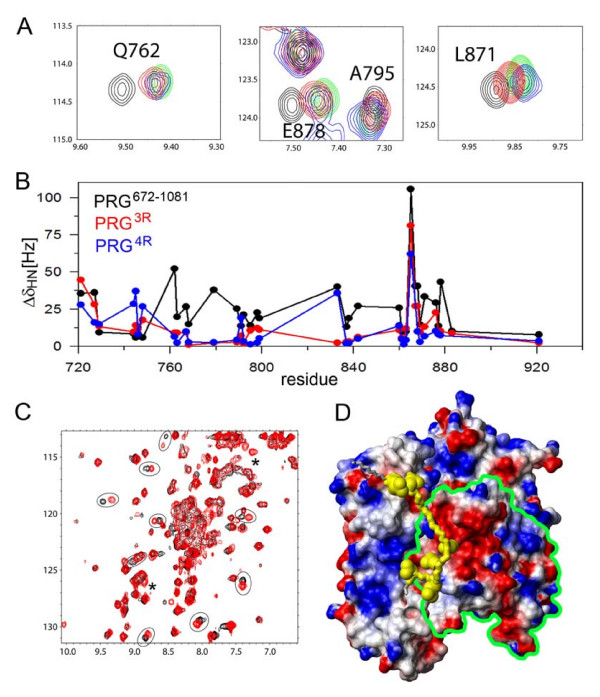
**The impact of mutations in the linker region on its interaction with the DH domain and with RhoA**. **(A) **comparison of 600 MHz ^1^H-^15^N TROSY-HSQC spectra of PRG^672–1081 ^(black), the two mutants: PRG^3R ^(red) and PRG^4R ^(blue) and DH-PH (green); **(B) **plot of chemical shift differences (Δσ_HN_) within DH domain induced by the wild-type linker (black), PRG^3R ^(red) and PRG^4R ^mutants (blue); (C) comparison of 600 MHz ^1^H-^15^N TROSY-HSQC spectra of ^2^H,^15^N-RhoA in complex with wild-type PRG^672–1081 ^(red) and PRG^4R ^(black); several amides with the largest chemical shift perturbations are circled, peaks labeled with asterisks could not be identified in the spectrum of PRG^4R^; (D) surface charge distribution calculated for the crystal structure of DH-PH/RhoA complex; the putative position of the linker is shown in yellow; side chains of acidic residues within the linker are shown in space filling representation; RhoA is delineated by a green boundary for clarity.

To understand how mutations within the linker modulate GEF activity, we measured NMR spectra of both the triple and quadruple charge reversal mutants of PRG^672–1081 ^(henceforth denoted PRG^3R ^and PRG^4R^) and analyzed the chemical shifts within the DH domains. We found that both mutants qualitatively show similar patterns of chemical shift perturbations as observed for the wild-type PRG^672–1081^. However, the magnitude of chemical shift changes is significantly decreased compared to the wild-type protein (Figs. 5A &5B). To quantify this effect, we calculated the total chemical shift change, Δσ_HN-total_, i.e. a sum of chemical shift changes observed for all amides assigned to the DH domain, compared to the isolated DH-PH tandem. For PRG^672–1081^, PRG^3R ^and PRG^4R ^the values of Δσ_HN-total _were 875, 480 and 404 Hz, respectively. Therefore, the mutations within the linker decreased the chemical shift changes in the DH domain by approximately 50% compared to the wild-type protein. This is consistent with the observed relief of the inhibitory effect, although it does not explain the enhanced catalytic activity of PRG^4R ^mutant.

The next step was to evaluate the effect of the mutations in the linker on the direct interaction of the PRG^712–1081 ^with RhoA. We measured ^1^H-^15^N TROSY-HSQC spectra of ^15^N-labeled RhoA in complexes with each of the two mutants, i.e. PRG^3R^, and PRG^4R^. The spectrum of RhoA in the presence of PRG^3R ^is essentially identical to that of RhoA in the presence of the wild-type PRG^672–1081 ^indicating no significant effect of the mutations in the linker on the interaction with RhoA (data not shown). However, noticeable changes are observed for RhoA in the presence of the PRG^4R ^with over a dozen resonances shifted by more than 25 Hz (Fig. 5C). As inferred from the NMR analysis, the linker of the PRG^4R ^mutant appears to interact with negatively charged surfaces of RhoA (Fig. 5D). Reversing the charge of acidic residues within the linker appears to have a pronounced effect on the kinetics of DH-PH/RhoA interaction and ultimately, the rate of nucleotide exchange.

### The role of PDZ-RGSL domains

To evaluate the impact of the mutations in the linker region on the autoinhibited core fragment of PRG containing all four functional domains, we carried out the nucleotide exchange assay for PRG^37–1081 ^with the D706R, E708R, E710R and D712R mutations. The rate of exchange was increased from ~20, in the wild-type PRG^37–1081^, to 43, i.e. only two-fold. Interestingly, this is equivalent to eliminating the PDZ and RGSL domains from wild-type PRG^37–1081^. This result shows that autoinhibition in the wild-type, full-length protein is an additive effect of a number of different interactions.

#### Functional assays are consistent with the in vitro model

**Figure 6:**
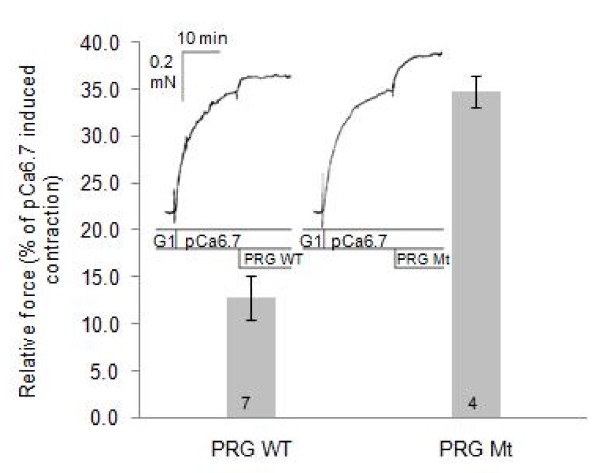
**Contractility assay**. Wild type (wt) and 4R mutant of the core fragment of PRG were added to β-escin permeabilized pulmonary artery precontracted with pCa 6.7 solution containing 1 μM calmodulin and 2 μM GTP. Concomitant increase in force is consistent with activation of the RhoA pathway. The 4R mutant, 10–15 μM, induced significantly greater Ca^2+^-sensitized force than the wild type protein (p = 0.002; n = 11. The magnitudes of the responses are normalized to pCa 6.7-induced force developed before addition of the recombinant PRG variants.

In order to assess if the *in vitro *studies are representative of biological properties of PDZRhoGEF in smooth muscle, we used a contractility assay of a permeabilized rabbit pulmonary artery (Fig. 6). In this assay, which measures force under isometric conditions, we are able to introduce recombinant protein directly into a smooth muscle strip, after contractile force is induced at pCa of 6.7. As we showed elsewhere [37], the addition of the wild-type recombinant DH-PH domain substantially increases the contractile force *via *RhoA activation. When the PRG^37–1081 ^fragment is added instead, the contractile force increases marginally, consistent with the autoinhibition of that fragment, while the same fragment with the mutations D706R, E708R, E710R, D712R, causes a significantly stronger contraction, albeit well below the DH-PH level, in full agreement with the *in vitro *assays.

**Figure 7:**
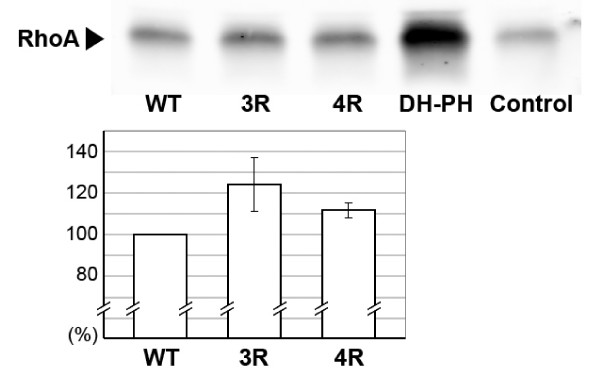
**Increase in the endogenous RhoA activation in NIH 3T3 cells monitored by the Rhotekin assay**. Upper panel: Rhotekin pull-down of RhoA•GTP from cells ectopically expressing full-length wild type (wt) PRG, the isolated DH-PH tandem and the 3R and 4R mutants of the full-length form. The cells were under serum free conditions. Bottom panel: quantitative assessment of RhoA activation using a set of three independent experiments. The isolated DH-PH tandem is not included because the expression level was significantly higher than that of the three full-length PRG variants, and while the increased endogenous RhoA activity as measured by the Rhotekin assay (see Methods Section), demonstrating that these expressed constructs are biologically active in cells. Mutant PRG-RhoGEFS were FLAG tagged. Western blotting for FLAG indicated that the mutants were expressed at an equal level while the DH-PH domain was overexpressed (data not shown) accounting for the greater level of active RhoA.

We then conducted a rhotekin-based assay to measure RhoA activation in NIH 3T3 cells transfected with full-length, wild-type human PRG, as well as 3R and 4R mutants of the full-length protein, and the isolated DH-PH tandem (Fig. 7). As expected, overexpression of the isolated DH-PH tandem resulted in significant increase in RhoA•GTP over the control. Lower but equal expression levels of wild-type and the 3R and 4R mutants in unstimulated serum starved cells resulted in greater Rho activation over control with the 3R and 4R having the greatest effect. These findings are consistent with significant autoinhibition of the full length PRG and also show that the 3R and 4R mutants are biologically active in cells.

### The model of autoinhibition

**Figure 8:**
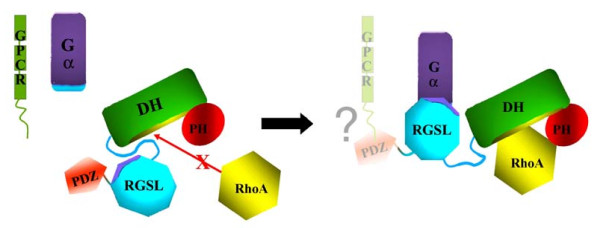
**A model of autoinhibition and activation of PDZRhoGEF**. In the inhibited protein the linker between the RGSL and DH domains is sequestered by the two modules, and the DH domain is not accessible to RhoA. Interaction with the Gα subunit, and possibly direct interaction with relevant GPCRs *via *the PDZ domain, relieve this inhibition by first dislodging the RGSL domain and then the inhibitory portion of the linker.

On the basis of the experiments described in this paper we propose a mechanism for the autoinhibition of PDZRhoGEF (Fig. 8). Unlike in other GEFs for which similar mechanisms were rationalized at the structural level, [11,12,40], the DH domain of PRG is not sterically obstructed by a single structural element, but instead several elements act synergistically within the autoinhibited, full-length protein. A fragment that spans all functional domains of PRG, i.e. PRG^37–1081^, retains only ~15% of the catalytic activity of the isolated DH-PH tandem, and so we concluded that intramolecular interactions within this fragment are primarily responsible for autoinhibition. This was expected, because RGSL and PDZ domains are thought to play a role in the autoinhibition/activation mechanism. Surprisingly, our *in vitro *catalytic assay revealed that removal of both of these domains results in only 2-fold increase in activity, indicating that other factors are involved. We traced the primary autoinhibitory element to a sequence motif upstream of the DH domain, harboring Asp706, Glu708 and Asp712, and Glu710. The PRG fragment encompassing residues 691 to 1081, i.e. with only 21 amino acids upstream of the DH domain, showed less than 50% of the activity of the DH-PH tandem. Replacing the four negatively charged residues with arginines not only abolished autoinhibition but generated a 3.3 fold enhancement of the nucleotide exchange activity, as compared to the isolated DH-PH tandem. However, when the mutations were introduced into the PRG^37–1081 ^core fragment containing all four functional domains, autoinhibition is largely restored, presumably by interactions mediated by the RGSL and/or PDZ domains which may stabilize the autoinhibitory element in its functional conformation in spite of the mutations.

Based on these results, we suggest that the activation of PRG, and probably also of the two other remaining members of this family of GEFs, i.e. LARG and p115, proceeds by a multistep mechanism. The primary autoinhibitory element resides upstream of the DH-PH tandem, but may not be accessible due to secondary interactions with RGSL and/or PDZ domains. This is why a conformational change involving the latter domains is *essential*, but not *sufficient *for full GEF activation. In agreement with this model, activated, GTP-bound Gα_13 _can bind directly to a fragment containing both the linker and DH-PH tandem of the p115 GEF, but without concomitant activation of the nucleotide exchange activity [41]. Thus, both the RGSL domain and the 'activation box' may act synergistically. Also, activation of GEF activity can be partially uncoupled from binding of Gα_13 _to the RGSL domain in p115 GEF: point mutations in the latter that reduce affinity for Gα_13 _have no effect on the activation of nucleotide exchange of p115 GEF [42]. Analogous mutants do not affect translocation of p115 GEF to particulate fractions in the presence of Gα_13_, suggesting that translocation of p115 GEF is due to complex mechanisms involving elements other than the RGSL domain [43].

## Conclusion

We here propose that in the closed, autoinhibited form, the RGSL domain interacts with both the RGSL-DH linker and the DH domain, or perhaps only with the linker, which serves as the direct inhibitory element (Fig. 7). When the RGSL domain binds to Gα_12/13_, the inhibitory element remains in place, but becomes exposed and accessible to secondary interactions which in consequence remove the linker from the functional DH surfaces. The dramatic effect of the charge reversal mutant also suggests that electrostatic interactions could play a major role, not only in relieving the autoinhibition, but also in positive regulation of the kinetics of RhoA binding and nucleotide exchange. It is typically assumed that the intrinsic catalytic properties of the DH domain, or more commonly the DH-PH tandem, reflect the maximum theoretical nucleotide exchange rate of the respective GEF. However, this is not always true. The isolated DH domain of Vav enhances the nucleotide exchange rate on Rac1 73-fold, but the addition of the PH-CRD fragment produces a further 13-fold enhancement [44]. Thus, it is possible that an activating interaction, e.g. involving Gα subunits, may not only relieve the autoinhibition, but also directly up-regulate the GEF activity. In that context, we note that the human Gα_12 _and Gα_13 _subunits differ with regard to their pI values, i,e. 9.8 and 8.2, respectively, and the positively charged surfaces could be important in the interaction with the DH/RhoA complex.

Finally, we note that the proposed mechanism might be relevant to all three GEFs of the RGSL-family. The amino acid sequences of both LARG and p115 show similar negatively charged patches in nearly identical locations: LARG contains a Glu-Asp-Glu tripeptide, while p115 GEF has an Asp-Glu-Gly-Glu motif (Additional File 1: Figure S2). Further work, underway in our laboratories, will hopefully establish the details of these pathways.

## Methods

### Protein Expression and Purification

Full-length and truncated versions of human PDZRhoGEF were PCR-amplified and subcloned into pDEST15 (Invitrogene) vector containing a glutathione S-transferase (GST) tag at the N-terminus, an rTEV protease site between GST and the insert, and a non-cleavable His_8 _tag at the C terminus. All constructs were verified by direct sequencing prior to protein expression. The proteins were expressed in the *E. coli *strain BL21 (DE3) RIPL (Strategene). Cell cultures were grown at 37°C in TB/ampicillin (100 μg/ml) and chloramphenicol (34 μg/ml), and induced with 1 mM isopropyl β-D-thiogalactopyranoside (IPTG) overnight at 18°C. Cell pellets were resuspended in 50 mM Tris pH 7.8, 300 mM NaCl, 0.5 mM EDTA, 1 mM dithiothreitol (DTT) (Buffer A), lysed using Sonifier 450 (VWR), and clarified by centrifugation at 40,000 × *g *for 45 min at 4°C. Supernatant was loaded on a glutathione-Sepharose 4B column (Pharmacia), pre-equilibrated with Buffer A. Protein was bound to the resin by gently rocking the column at 4°C for 1 hour and eluted with 20 mM glutathione in 50 mM Tris, pH 8.0, 50 mM NaCl, 0.5 mM EDTA, 1 mM DTT (Buffer B). Fractions containing pure fusion protein were pooled, digested overnight with rTEV protease in the same buffer and then dialyzed against Buffer B to remove glutathione. After rTEV cleavage, the protein solution was loaded again on glutathione-Sepharose 4B column and rocked for 1 hour to remove GST and the uncut fusion protein. Flow-through was collected and diluted with 50 mM Tris pH 8.0, 150 mM NaCl (Buffer C). The solution was then loaded onto Ni-NTA column pre-equilibrated with buffer C and eluted with 200 mM imidazole in Buffer C. Fractions containing pure protein were pooled and dialyzed against 20 mM Tris pH 7.5, 150 mM NaCl, 1 mM DTT. Pure protein was concentrated and stored at -80°C for the fluorescence assay.

Proteins labeled with stable isotopes (^2^H, ^13^C, ^15^N) were expressed in minimal media with (^15^NH_4_)_2_SO_4 _as sole source of nitrogen and ^13^C- or ^2^H,^13^C-glucose as a source of carbon. The media were enhanced by the addition of labeled BioExpress (Cambridge Isotope Labs.). Four types of labeling scheme were used: ^15^N, ^13^C ^15^N, ^2^H ^15^N and ^2^H ^13^C ^15^N.

The expression and purification of human RhoA^F25N ^(residues 1–181) was carried out as described previously [45].

### Guanine Nucleotide Exchange Assay

Fluorescence spectroscopy analysis of *N*-methylanthraniloyl (mant)-GTP incorporation into RhoA was carried out using a FluoroMax-3 (Jobin Yvon Inc.) spectrofluorimeter. The exchange reaction was carried out at 22°C for 5 min in a 1.5 ml Eppendorf tube containing 1 μM GDP-preloaded RhoA and 0.5 μM mant-GTP in 20 mM Tris-HCl, (pH 7.5), 50 mM NaCl, 5 mM MgCl_2 _and 1 mM DTT. After equilibration, assayed proteins were added at 0.1 μM concentration and increases in mant-GTP fluorescence was monitored (λ_ex _= 356 nm, λ_em _= 450 nm). The control experiment, in the absence of other proteins, reflects intrinsic exchange activity of RhoA, measured after equivalent equilibration time. Each exchange experiment was carried out three times independently. The initial rates of guanine nucleotide exchange were determined by regression analysis. The average variation among measured rate did not exceed 10%. Fold stimulation values were calculated as the ratio of the initial exchange rate of DH-PH-stimulated reaction to the intrinsic rate of exchange for the wild type RhoA.

### Optimization of DH-PH samples for NMR experiments

In order to optimize both the stability of protein samples and the sensitivity of NMR experiments, we screened a large number of different buffers. We prepared 0.5 mM solution of DH-PH in several buffers with varied salt concentrations and kept the samples at 4 – 35°C with periodic monitoring of the concentration and the level of sample degradation using SDS-PAGE. We found the best solubility and stability of DH-PH samples in 50 mM TRIS buffer, pH 7.5 and 150 mM NaCl. However, such conditions do not provide optimal sensitivity for cryogenic probes due to high salt concentration [46]. Therefore, to achieve the highest possible sensitivity of NMR experiments we tested several salt-free buffers and found a mixture of 200 mM MOPS/TRIS, pH 7.5, to be optimal. In such conditions DH-PH can remain in solution at a concentration above 0.3 mM for over a week at 25°C. Higher temperature, ~30°C, reduces the lifetime of the sample to approximately 3–4 days.

Because backbone assignment of perdeuterated proteins depends on efficient back-exchange of amide protons, we dissolved ^15^N-labeled DH-PH in D_2_O based buffer and monitored H/D exchange using the HSQC spectra. We observed complete exchange of amide protons over one week.

### Chemical shift assignment

To assign the backbone amide chemical resonances of isolated DH and PH domains, we used standard triple resonance experiments (HNCO, HNCA, HN(CO)CA, HNCACB, CBCA(CO)NH and 3D ^15^N-edited NOESY with 200 ms mixing time). The spectra for 0.3 mM ^2^H,^13^C,^15^N-labeled DH domain in 200 mM MOPS/TRIS buffer, pH 7.5, and 2 mM DTT were collected using Varian Inova 600 MHz at 25°C. The spectra for ^13^C,^15^N-labelled PH domain were recorded using Varian Inova 500 MHz for 0.5 – 0.7 mM protein concentrations in 600 mM NaCl, 50 mM TRIS pH 7.0 and 2 mM DTT buffer at 25°C.

To assign the resonances in the intact DH-PH tandem, we used ^2^H,^13^C,^15^N labeled protein at 0.3–0.4 mM concentration in 200 mM MOPS/TRIS buffer at pH 7.5 and 2 mM DTT. The NMR spectra were collected at 30°C using Varian Inova 800 MHz spectrometer equipped with cryogenic probe. The following TROSY-based experiments were conducted: TROSY-HNCA, TROSY-HNCO, 3D ^15^N-edited TROSY-NOESY with 250 ms mixing time.

### Chemical shift perturbation experiments

In order to analyze chemical shift perturbations we measured series of experiments using Varian Inova 900 MHz, Varian Inova 600 MHz and Bruker Avance 600 MHz spectrometers equipped with cryogenic probes. Samples for NMR contained 0.2 to 0.3 mM protein concentrations in 200 mM MOPS/TRIS buffer, pH 7.5 with 1 mM DTT. All of the ^1^H-^15^N TROSY-HSQC spectra were collected at 25°C.

### Tissue Preparation and Force Measurements

Rabbits were anesthetized and killed with isoflurane. Small strips (100–150 μm thick, 150–200 μm wide, and 2–3 mm long) of rabbit pulmonary artery were dissected and freed from connective tissue. Single strips were tied with monofilament silk to the fine tips of two tungsten needles, one of which was connected to a force transducer and mounted in a well on a bubble plate, as described previously [47]. After measuring contractions induced by high 154 mM K^+^, the strips were incubated at room temperature (21–22°C) in Ca^2+ ^free relaxing solution (G1) containing 4.5 mM MgATP and 1 mM EGTA for several minutes and permeabilized with 75 uM β-escin for 25 min. This permeabilization protocol permits penetration of molecules up to 150 kDa [48]. To deplete the sarcoplasmic reticulum of calcium [47]. and inhibit NO-synthase activity, every strip was also treated with 10 μM A23187 (Calbiochem) and 10 μM L-NAME (Sigma) (for additional 10 min in the G1 solution. Buffer in protein samples of wild type and 4R mutant PRG core fragment (PRG^37–1081^) was exchanged against pCa 6.7, 1 μM calmodulin and 2 μM GTP. Samples of ~10–15 μM of recombinant protein were used to stimulate the force responses.

### The Rhotekin assay

The cDNA of full-length wild-type PDZ-RhoGEF, as well as 3R and 4R mutants and the isolated DH-PH tandem were PCR amplified with flanking restriction sites and introduced into p3XFLAG-myc-CMV-24 mammalian expression vector (Sigma-Aldrich, St. Louis MO). NIH 3T3 cells were cultured on a 10 cm culture plate in Dulbecco's Modified Eagle Medium (DMEM; Invitrogen-Gibco) supplemented with 10% fetal bovine serum (FBS; Invitrogen-Gibco) at 37°C in 5% CO_2_. The plasmids for the ectopic expression with N-terminal 3XFLAG and C-terminal myc-tag, and coding sequences for the full-length wild-type, 3R or 4R mutant PDZ-RhoGEF or the DH-PD tandem were transfected into cells using Lipofectamine 2000 (Invitrogen) following manufacture's standard protocol. The cells were treated for transfection, cultured overnight, treated for transfection for the second time and cultured in DMEM without FBS, changed 8 hours after the second transfection, overnight. RhoA activation was determined by precipitation of active GTP-bound RhoA (RhoA•GTP) with a glutathione S-transferase (GST)-fusion protein of the Rho-binding domain (RBD) of the Rho effector rhotekin as previously described [49]. Briefly, the cells on a 10 cm plate were lysed in 500 μl of ice cold lysis buffer (25 mM HEPES, pH 7.5; 150 mM NaCl; 1% NP-40; 10 mM MgCl2; 1 mM EDTA; 6% Glycerol; 1% Protease inhibitor cocktail (Sigma-Aldrich, St. Louis MO)), and cleared by centrifugation at 14,000 g for 10 minutes at 4°C. Ectopic expression of full-length wild-type, 3R and 4R mutant PDZ-RhoGEF at an equal level and the DH-PD tandem over-expression were confirmed by subjecting the supernatant to Western blotting for FLAG epitope tag (data not shown). The supernatant was added to GST-RBD agarose beads (Millipore, Billerica MA) and affinity precipitated active RhoA•GTP was detected by Western blotting for RhoA using a mouse monoclonal antibody (Santa Cruz Biotechnology Inc., Santa Cruz CA). Detection and quantification of the signal was performed using the Odyssey system software (Li-Cor, Lincoln NE).

## Authors' contributions

MZ expressed and purified all protein constructs and conducted the nucleotide exchange assays. TC conducted the NMR experiments, analyzed the data and drafted relevant sections of the manuscript. KM carried out the Rhotekin assay. MVA carried out force measurements. UD, JHB, AVS and ZSD conceived the project, analyzed the data and drafted the manuscript. All authors read and approved the final manuscript.

## Supplementary Material

Additional file 1 **Supplementary figures**. **Figure S1 **Coomassie stained SDS-PAGE gel illustrating the purity of protein samples used for functional studies. **Figure S2 **Sequence alignment of the putative regulatory motifs upstream of the DH domains of PRG, LARG and p115 guanine exchange factors for RhoA. Click here for file
