# Supplementary figures and images for: On the mechanism of autoinhibition of the RhoA-specific nucleotide exchange factor PDZRhoGEF
Source: BMC Struct Biol. 2009 May 21;9:36. doi: 10.1186/1472-6807-9-36 (PMC2695464; doi:10.1186/1472-6807-9-36)

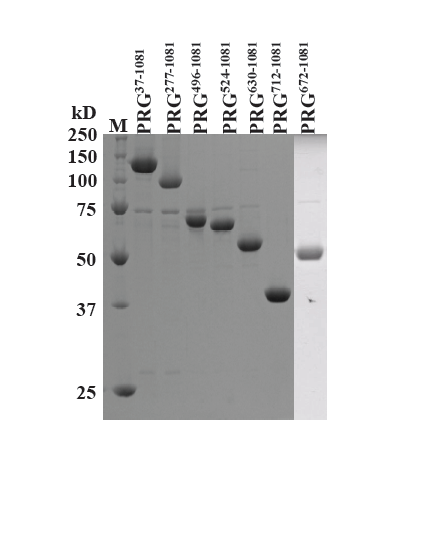


**Figure S1**

**Figure S2**

Supplement: Additional file 1 — Supplementary figures. Figure S1 Coomassie stained SDS-PAGE gel illustrating the purity of protein samples used for functional studies. Figure S2 Sequence alignment of the putative regulatory motifs upstream of the DH domains of PRG, LARG and p115 guanine exchange factors for RhoA. [file 1472-6807-9-36-S1.doc]
